# The Detection and Characterization of Herpes Simplex Virus Type 1 in Confirmed Measles Cases

**DOI:** 10.1038/s41598-019-48994-5

**Published:** 2019-09-04

**Authors:** Chongshan Li, Yunyi Li, Yuying Yang, Jing Wang, Caixia Zhu, Suwen Tang, Cong Pang, Wei Tang, Qiliang Cai, Zhi Li, Jiayu Hu, Xiaoxian Cui, Xi Zhang, Songtao Xu, Yan Zhang, Zhengan Yuan, Yunwen Hu, Zhenghong Yuan

**Affiliations:** 1 Scientific Research Unit, Shanghai Public Health Clinical Center, Fudan University, Shanghai, 201508 People’s Republic of China; 2 Division of Microbiology, Shanghai Municipal Center for Disease Control and Prevention, Shanghai, 200336 People’s Republic of China; 3 MOE & MOH Key Laboratory of Medical Molecular Virology, School of Basic Medicine, Shanghai Medical College, Fudan University, Shanghai, 200032 People’s Republic of China; 4 NHC Key Laboratory of Medical Virology and Viral Diseases (National Institute for Viral Disease Control and Prevention, Chinese Center for Disease Control and Prevention), Beijing, 102206 People’s Republic of China; 5 Shanghai Municipal Center for Disease Control and Prevention, Shanghai, 200336 People’s Republic of China

**Keywords:** Herpes virus, Measles virus

## Abstract

Based on measles surveillance in Shanghai, People’s Republic of China, from 2006 to 2015, we found that measles virus isolates from 40 throat swab samples exhibited atypical cytopathic effects in Vero/hSLAM cells, which was found to be a result of coinfection with measles virus (MeV) and human herpes simplex virus type 1 (HSV-1). Serological and molecular approaches were used to confirm and characterize the coinfections in these patients. Among the 40 measles cases, measles-specific IgM was detected in 37 cases, while measles-specific IgG was detected in 27 cases. HSV-1-specific IgM and IgG were detected in 7 and 34 cases, respectively, suggesting that most of the MeV infections were primary, but that HSV-1 infection was due to the reactivation of latent virus in most cases. The titers of HSV-1 IgG in patients with either measles or measles-HSV-1 coinfection were significantly higher than those in the healthy group (P = 0.0026 and P < 0.0001, respectively); however, there was no significant difference in the titers of HSV-1 IgG in the MeV and MeV-HSV-1 coinfection patients (P = 0.105). Nucleic acids from MeV and HSV-1 were detected in 40 and 39 throat swabs, respectively. Twenty five MeV RNA sequences were genotyped, and all represented genotype H1, which is the endemic genotype in China. Sequences from the glycoprotein G gene of HSV-1 were used to classify the isolates into two distinct phylogenetic groups: 34 belonged to group A and 3 belonged to group B.

## Introduction

Measles is a common childhood infection with clinical symptoms that includes fever, maculopapular rash, cough, coryza and conjunctivitis. The measles virus (MeV) causes measles and belongs to the genus *Morbillivirus* in the family *Paramyxoviridae*. The genome of MeV is a negative-sense, single-stranded RNA that contains eight genes (N, P/V/C, M, F, H, and L) that encode six proteins. There is only one measles serotype. To monitor the progress of measles elimination, molecular surveillance has been implemented in most member states by the World Health Organization (WHO). The 450 nt sequence that encodes the C-terminus of the N protein (N-450) is the standard genotyping window used for genotyping MeV. Currently, wild-type MeV is divided into 24 genotypes^1–3^.

In China, measles surveillance began in 1993. To enhance measles surveillance, a laboratory surveillance network was established in 2001. To date, genotype H1 remains an endemic genotype in China, and subgenotype H1a has been the predominantly circulating strain since 2005^4^. Other genotypes, including H2, D9, D4, D8, and B3, were also detected in imported cases^5–9^. In Shanghai, high-quality measles surveillance has been maintained since 2001. The measles surveillance system (MSS) detected the first importations of MeV genotypes D8 in 2012^7^ and B3 in 2013^9^ into China.

Suspected measles cases can be confirmed by the detection of measles-specific immunoglobulin M (IgM) antibodies or viral RNA. Isolation of MeV from clinical specimens using cell lines susceptible to wild-type MeV infection (e.g., Vero/hSLAM cells) can also be used as a confirmatory test^10^. Viral nucleotide sequences can be obtained from clinical specimens and/or viral isolates. MeV infection can cause cell membrane fusion and induce the formation of multinucleated giant cells (syncytia) in susceptible cells^11^. Such a pronounced cytopathic effect (CPE) is often used to indicate the presence of MeV in cell culture. During serial passages of cultures from a throat swab specimen from a suspected measles case, we observed changes in the types of CPE observed in infected Vero/hSLAM cells. Syncytia were observed in cells inoculated with the first two passages of virus; however, beginning with the third passage, the infected cells exhibited cell rounding with a decrease in syncytia. Human herpes simplex virus type 1 (HSV-1) infection was confirmed in this culture by real-time PCR. A retrospective study was conducted to investigate coinfection of MeV and HSV-1 based on samples collected from 2006 to 2015. Forty coinfected cases were identified from a total of 4921 cell cultures obtained from throat swabs.

## Results

### Measles surveillance in Shanghai from 2006 to 2015

From 2006 to 2015, a total of 7,276 measles cases were confirmed in Shanghai. The measles incidence was between 0.35/100,000 and 6.79/100,000 annually. The ratio of male to female cases was 1.28:1. Most cases occurred in populations aged 8–11 months, 20–29 years and 30–39 years. Adults aged >20 years accounted for 50% of all cases. Measles immunization has been documented since 2009 when the MSS was established. The majority of measles cases occurred in individuals without an immunization history or who had an unknown history. According to the MSS, a total of 186 cases were identified in individuals who had received at least one dose of measles-containing vaccine (MCV), who accounted for 2.48% to 13.25% of the cases between 2009 and 2015. Among these cases, 70.43% had received one dose of MCV, 24.73% had received two doses of MCV, and 4.84% had received more than two doses of MCV.

From 1253 MeV isolates during this period, 40 cases of MeV and HSV-1 coinfection were identified. The average age was 23 (the age ranged from 6 months to 53 years old). Twenty-four were adults older than 20 years old. Three had documented MCV (SH11300, SH15370, and SH15185), whereas the remaining had an unknown or no history of MCV or disease (Table 1). All 40 patients reported fever and rash, 33 (82.5%) experienced coughing, and 24 (60%) experienced catarrhal symptoms.

**Table 1:** Demographic information and test results for patients coinfected with MeV and HSV-1 from 2006 to 2015.

Sample ID	Gender	Age(Y)	MCV dose	real-time PCR		IgM		IgG	
				MeV	HSV-1	MeV	HSV-1	MeV	HSV-1
SH08035	M	1.2	N/A	Pos	Pos	Pos	Neg	Neg	Neg
SH11300	M	0.8	1	Pos	Pos	Neg	Neg	Neg	Neg
SH12538	M	1.2	0	Pos	Pos	Pos	Neg	Neg	Neg
SH13774	F	0.5	0	Pos	Pos	Pos	Neg	Neg	Pos
SH06064	F	4	N/A	Pos	Pos	Pos	Neg	Pos	Neg
SH151187	F	50.3	N/A	Pos	Pos	Pos	Neg	Pos	Neg
SH12244	M	31.4	N/A	Pos	Pos	Pos	Neg	Neg	Pos
SH13017	M	45.9	N/A	Pos	Pos	Pos	Neg	Neg	Pos
SH13143	F	38.1	N/A	Pos	Pos	Pos	Neg	Neg	Pos
SH13176	M	33.8	N/A	Pos	Pos	Pos	Neg	Neg	Pos
SH13294	F	46	N/A	Pos	Pos	Pos	Neg	Neg	Pos
SH13770	F	50	N/A	Pos	Pos	Pos	Neg	Neg	Pos
SH15679	F	30	N/A	Pos	Pos	Pos	Neg	Neg	Pos
SH07040	M	5.2	N/A	Pos	Pos	Pos	Neg	Pos	Pos
SH11303	M	15.8	N/A	Pos	Pos	Pos	Neg	Pos	Pos
SH12164	M	29.8	N/A	Pos	Pos	Pos	Neg	Pos	Pos
SH12354	M	32.7	N/A	Pos	Pos	Pos	Neg	Pos	Pos
SH12525	M	44.9	N/A	Pos	Pos	Pos	Neg	Pos	Pos
SH12532	M	34.3	N/A	Pos	Pos	Pos	Neg	Pos	Pos
SH12649	M	23.8	N/A	Pos	Pos	Neg	Neg	Pos	Pos
SH13027	M	35.6	N/A	Pos	Pos	Pos	Neg	Pos	Pos
SH13073	M	22	N/A	Pos	Pos	Pos	Neg	Pos	Pos
SH13390	M	25	N/A	Pos	Pos	Pos	Neg	Pos	Pos
SH13401	F	19.4	0	Pos	Pos	Pos	Neg	Pos	Pos
SH13577	M	34.9	N/A	Pos	Pos	Pos	Neg	Pos	Pos
SH13659	M	32.6	N/A	Pos	Pos	Pos	Neg	Pos	Pos
SH14354	M	27.5	N/A	Pos	Pos	Pos	Neg	Pos	Pos
SH14622	M	27.3	N/A	Pos	Pos	Pos	Neg	Pos	Pos
SH151195	M	0.7	N/A	Pos	Pos	Pos	Neg	Pos	Pos
SH15180	F	40.8	0	Pos	Pos	Pos	Neg	Pos	Pos
SH15214	M	1	0	Pos	Pos	Pos	Neg	Pos	Pos
SH15334	F	32.5	N/A	Pos	Pos	Pos	Neg	Pos	Pos
SH15370	M	12.1	2	Pos	Pos	Neg	Neg	Pos	Pos
SH12441	M	1	0	Pos	Pos	Pos	Pos	Neg	Pos
SH15185	M	1.5	1	Pos	Pos	Pos	Pos	Neg	Pos
SH06036	M	2	N/A	Pos	Neg	Pos	Pos	Pos	Pos
SH06076	F	1.7	N/A	Pos	Pos	Pos	Pos	Pos	Pos
SH09046	M	1.1	N/A	Pos	Pos	Pos	Pos	Pos	Pos
SH14297	M	31	N/A	Pos	Pos	Pos	Pos	Pos	Pos
SH15226	M	52.9	N/A	Pos	Pos	Pos	Pos	Pos	Pos

### Detection of viral nucleic acids

Viral nucleic acids were detected by real-time PCR/RT-PCR from throat swab samples or infected Vero/hSLAM cultures, which were divided in to 3 groups, the coinfection group (n = 40), measles group (n = 463) and healthy group (n = 192). Among the 40 MeV and HSV-1 coinfection cases, MeV RNA was detected in 40 (100.0%) throat swabs by RT-qPCR. There were no significant differences in the MeV RNA copy numbers between the coinfection group and the measles group (P = 0.5033). HSV-1 nucleic acids were detected in 39 of the 40 throat swabs by qPCR, and the copy number of HSV-1 DNA in the coinfection group was significantly higher than that in the measles group (P = 0.0028).

Of the 463 throat swabs from MeV cases, 17 (3.67%) were positive for HSV-1 DNA, whereas 3 (1.56%) swabs from the healthy group were positive. There was no significant difference in the positive HSV-1 DNA rate between these two groups (P = 0.1316).

### Serological results

Serum samples from the 40 MeV and HSV-1 coinfection cases were tested for IgM and IgG antibodies (Table 1), the OD value of IgM and IgG and judgement were supplied in detail as Supplementary Table S1. Thirty-seven (92.5%) were positive for MeV IgM, and 27 (67.5%) were positive for MeV IgG. Although three (SH11300, SH12649, and SH15370) were negative for MeV IgM, MeV RNA was detected in the corresponding throat swabs. Of the 40 cases, seven (17.5%) and 34 (85.0%) were positive for HSV-1 IgM and IgG, respectively. Five (SH06064, SH08035, SH11300, SH12538 and SH151187) were negative based on both HSV-1 IgG and IgM. Among these five patients, four were children under 5, while SH151187 represented a patient older than 50. Table 2 shows the titers of HSV-1 IgG antibody in the healthy group, coinfection group, and measles group. Statistically significant differences were found between the measles group and the healthy group (P = 0.0026), the healthy group and the coinfection group (P < 0.0001), but no significant difference was found between the measles group and the coinfection group (p = 0.105).

**Table 2:** Titers of HSV-1 IgG antibody in different groups.

Population group	No. of samples	No. of positive samples	Positive rate (%) (95% CI)	GMT (U/mL) (95% CI)
measles group	741	540	72.87 (69.67–76.08)	58.75 (55.55–62.14)
healthy group	741	424	57.22 (53.66–60.78)	43.20 (39.85–46.83)
coinfection group	40	34	85.00 (73.93–96.07)	72.20 (54.63–95.44)

### Virus isolation and laser scanning confocal microscopy results

A total of 1253 MeV isolates were obtained from 4921 throat swabs between 2006 and 2015. The proportion of MeV and HSV-1 coinfection ranged from 0.0–11.1% each year. All MeV isolates produced syncytium formation in infected Vero/hSLAM cells during the first three passages. Forty MeV isolates exhibited another type of CPE characterized by larger and rounder cells in cultures as early as the 3^rd^ passage, which became more prominent in the subsequent passages. These cultures were confirmed to be coinfected with MeV and HSV-1 by the detection of viral nucleic acids.

**Figure 1:**
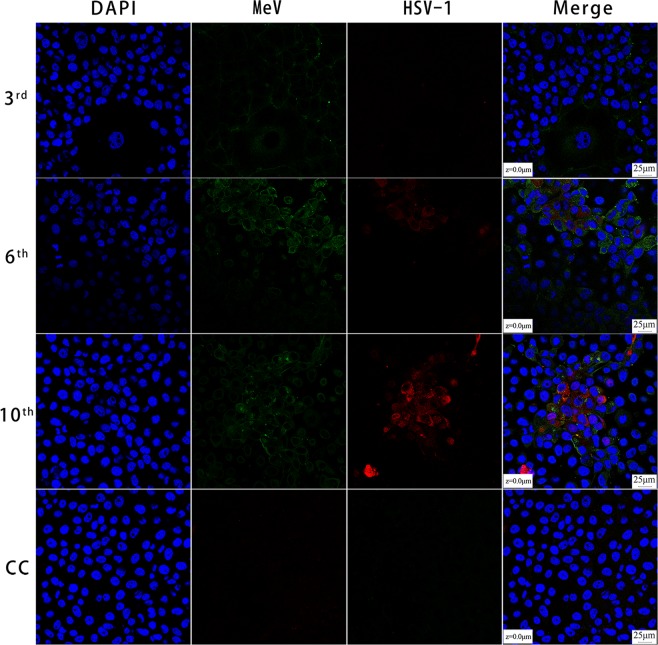
Laser scanning confocal microscopy of Vero/hSLAM cells coinfected by MeV and HSV-1 from different passages. Vero/hSLAM cells were infected with virus isolated from the clinical throat swab from SH13390 at the third, sixth, and tenth passage; these cells and a cell control (3^rd^, 6^th^, 10^th^, CC) as indicated on the left were subjected to immunostaining using antibodies against MeV M (green) and HSV-1 ICP0 (red) protein. Images were captured at 40× magnification using a Leica TCS SP8 confocal microscope (Leica Microsystems; Wetzlar, Germany). The cell nuclei were stained with DAPI.

Laser scanning confocal microscopy was used to demonstrate the dynamics of MeV and HSV-1 infection in coinfected Vero/hSLAM cells (Fig. 1). In cells infected with SH13390 at passage 3, MeV infection, detected by antibody of anti-MeV matrix (M) protein, as indicated by green fluorescence, was readily observed in some cells, whereas very few HSV-1-infected cells, detected by antibody of anti-HSV-1 ICP0, as indicated by red fluorescence, were found. In the 6^th^ passage of the infected culture, significant increases in MeV and HSV-1 infection were observed. In HSV-1-infected cells, the red fluorescence appeared as granules in the cytoplasm. As passaging continued, the number of cells stained with red fluorescence increased, while the number of cells stained with green fluorescence decreased. Eventually, at the 17^th^ passage, only cells with red fluorescence were detected (data not shown).

### Sequence analysis of MeV and HSV-1 isolates

**Figure 2:**
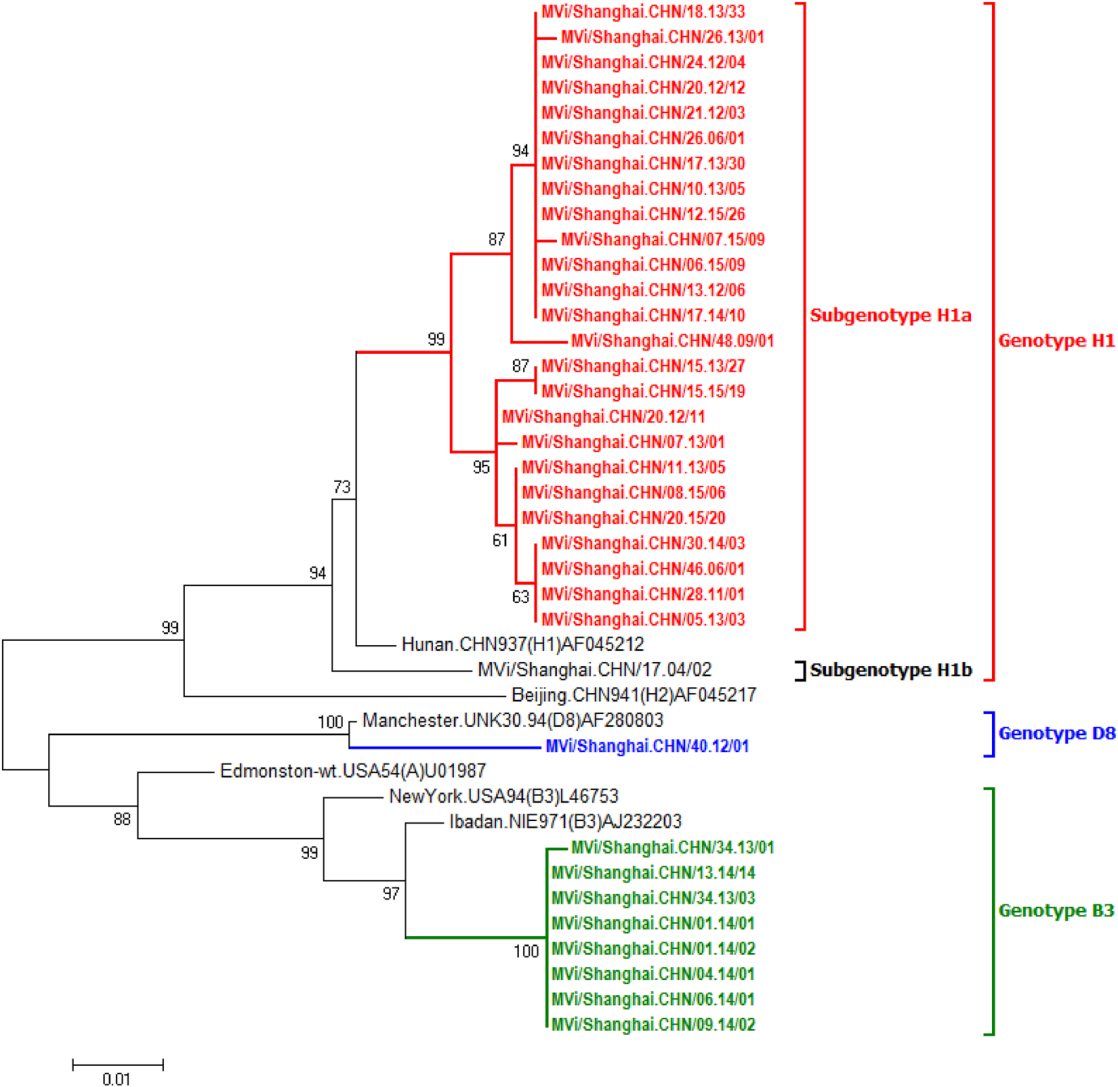
Phylogenetic tree of selected MeV strains isolated from Shanghai from 2006–2015 based on the N-450 sequences. Sequences shown in color are from this study: red represents HSV-1 coinfection cases, blue represents genotype D8 and green represents genotype B3. Sequences in black are from reference strains of different genotypes or subgenotypes. The numbers at the nodes are bootstrap values.

Of the 1,213 MeV isolates from the measles group (excluding the 40 MeV isolates from MeV and HSV-1 coinfection cases), 1,204 were H1a subgenotype of genotype H1, one was genotype D8, and eight were genotype B3. Genotyping of 25 MeVs from the coinfection cases was achieved by amplifying the MeV RNA directly from the throat swab samples (GenBank accession numbers: MN166369-MN166392, MN226847-MN226857; More sequence information of MeV isolates were provided as Supplementary Table S2), and all were genotype H1a (Fig. 2). The nucleic acid sequences of these 25 MeV exhibited 97.8–100.0% identity, and there was 98.0–100% amino acid sequence identity. The nucleotide sequence identity among the sequences of the MeV viruses and the reference sequence of genotype H1 (GeneBank: AF045212) was 97.3–98.0% (96.0–98.0% for the amino acid sequences). The overall nucleotide sequence identity between these 25 MeV strains and the 27 MeV reference sequences for all other genotypes (excluding genotype H1) was 88.3–93.6% (86.7–93.3% amino acid sequence identity).

**Figure 3:**
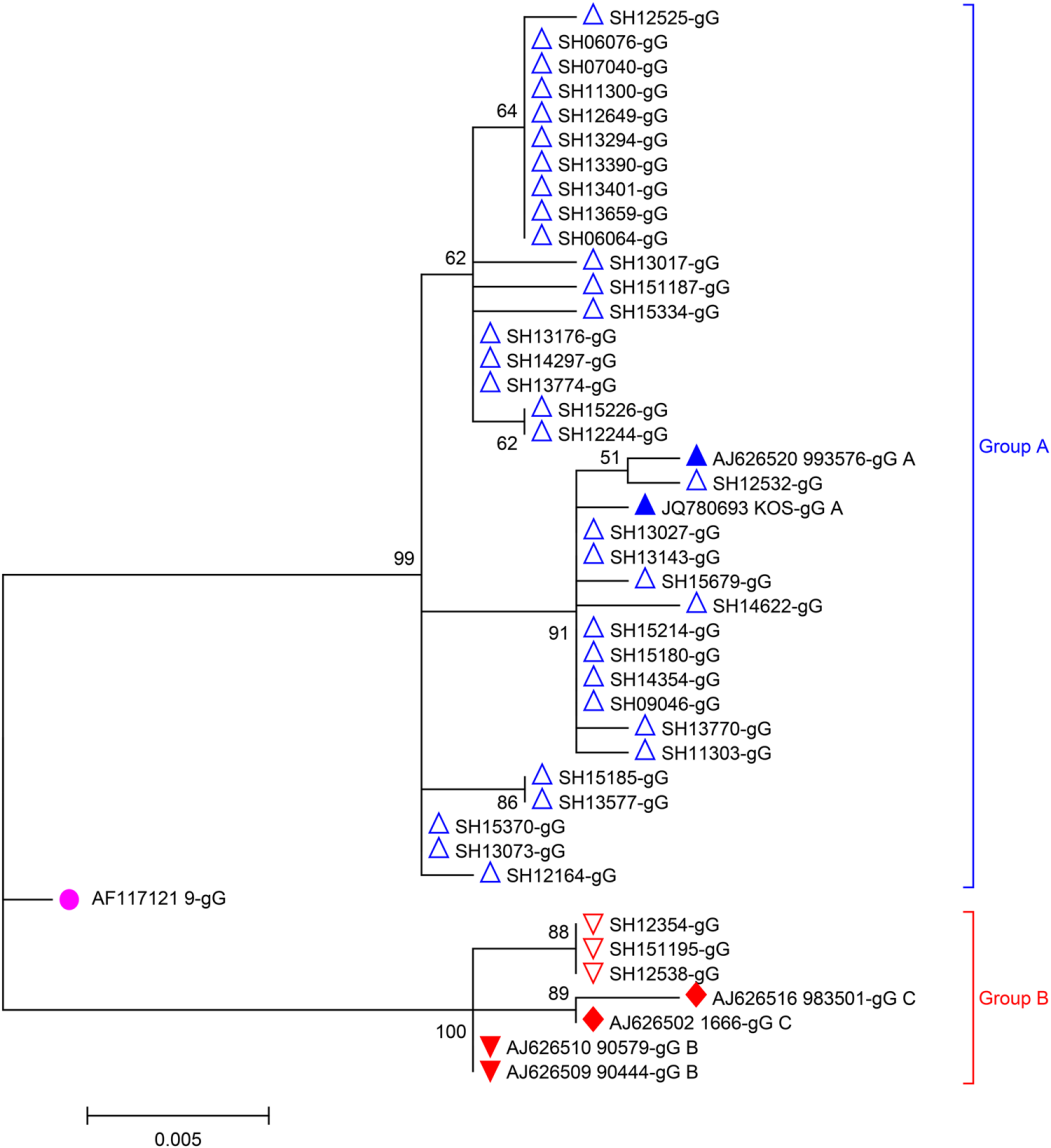
Phylogenetic tree of HSV-1 isolates from Shanghai from 2006–2015 based on nucleotide sequences from the gG gene. : group A isolates from coinfection cases; : reference strains from group (A); : group B isolates from coinfection cases; : reference strains from group B; : reference strains from group C; : AF117121 (a recombinant strain reported previously^28^). The numbers at the nodes are bootstrap values.

We sequenced 37 HSV-1 isolates from the 40 coinfected cultures (GenBank accession numbers: MN166405-MN166441; More sequence information of HSV-1 isolates were provided as Supplementary Table S3). All contained 4–10 guanine-adenine-guanine (GAG) trinucleotide tandem repeats beginning at nt 235 (GenBank: JN555585, using the same numbering scheme) in the gG region. Point mutations in the GAG repeats were detected in some viruses. Based on phylogenetic analysis, these 37 HSV-1 isolates were divided into two lineages. The majority (34/37) of the sequences belonged to group A, and the remaining sequences belonged to group B (Fig. 3). The group A HSV-1 isolates had 98.8–100.0% nucleic acid identity and 97.0–100.0% amino acid sequence identity, while all three group B isolates had identical sequences. The nucleic acid identity between groups A and B was 96.5–97.3%, and the amino acid sequence identity was 94.0–95.7%. In the region between nt 267–703, the viruses in group A had completely different bases than the viruses in group B at 17 loci (Table 3). Ten of these 17 loci occurred at the first or second positions of codons, resulting in missense substitutions in the encoded protein. No recombination event was detected in the gG gene in any of the 37 isolates.

**Table 3:** Analysis of the loci in recombined regions in the HSV-1 gG gene.

Nucleotide site	267	324	331	342	344	348	350	385	392	429	449	471	482	487	498	620	703
Group A	T	T	G	C	G	T	A	C	G	A	G	A	A	C	G	T	C
AF117121 9-gG	C	T	G	C	G	T	A	C	G	A	T	C	G	A	A	G	T
Group B	C	C	T	T	A	C	T	T	A	C	T	C	G	A	A	G	T
Group A			Val		Gly		Asp	Pro	Gly		Ser		Gln	Pro		Val	Pro
Group B			Phe		Glu		Val	Ser	Asp		Ile		Arg	Thr		Gly	Ser

The nine loci between nt 324 and nt 429 were those where recombination occurred between groups A and B in AF1171219.

## Discussion

We conducted a retrospective study to examine HSV-1 infection in confirmed MeV cases in Shanghai from 2006 to 2015. Coinfections of MeV with other pathogens, such as adenovirus, parainfluenza virus, parvovirus B19, rubella virus, HHV-6, human immunodeficiency virus (HIV), metapneumovirus and *Streptococcus pneumoniae*, have been reported^12–14^. However, coinfection of MeV with HSV-1 has not been reported elsewhere. HSV-1 is commonly associated with HSV-2 and HIV infection, and coinfection of Lyme borreliosis and HSV-1 has also been reported^15–17^. In addition, since both HSV and VZV can establish latent infections in sensory neurons after primary infection, a rare case of coinfection due to the reactivation of both HSV and VZV has been reported^18^. In this study, nucleic acids from the rubella virus, dengue fever virus, EBV, B19, Adv, HEV, HHV6, CMV or VZV were not detected in any of the clinical throat swabs or cell cultures from the measles or coinfection groups.

Based on the serological testing and immunization records, the majority of the codetection cases (37/40, 92.5%) were primary measles infections. SH11300, SH15185 and SH15370 had received at least one dose of MCV. SH11300 was negative for MeV IgM and IgG. This case had received a dose of MCV 5 days before the onset of rash. Unfortunately, we were not able to determine whether the infection was a coincidental event or a vaccine-associated measles case due to a lack of sequence information. SH15370, which was only positive for MeV IgG, was classified as secondary vaccine failures. SH15185, which was only positive for IgM and the patient was 1.5 years old, might be a primary vaccine failure^19,20^. SH12649, which was lack of immunization records and positive only to IgG test, couldn’t be confirmed as immune failures, suggesting prior exposure to MeV or vaccine. In the same way, there were 25 cases testing positive to both IgM and IgG and it may be due to the window period of immunological test under primary infections^21^. SH11300, SH12649 and SH15370 were MeV IgM negative but were confirmed measles cases based on RT-qPCR testing. Sera were collected from these three cases within 2 days after the onset of rash.

The rates of HSV-1 IgG detection in the coinfection (85%) and measles groups (73%) were significantly higher than in the healthy group (57%). The HSV-1 IgG detection rates are lower than the reported seroprevalence (92%) in China^22^. Among the 40 coinfection patients, serological results suggested that 13 of these cases were likely to be primary infections of HSV-1, and 10 of these patients were under the age of 5. Upon comparison of the HSV-1 IgG titers, the titers in the MeV group and the coinfection group were significantly higher than in the healthy group. In addition, we noticed that the HSV-1 DNA copy number in the coinfection group was higher than that in the MeV group, whereas no significant difference was found in the amounts of MeV RNA measured in patients with or without HSV-1 coinfection. Thus, coinfection with HSV-1 does not affect the shedding of MeV based on the throat swabs; however, MeV infection may have some impact on HSV-1 infection and/or reactivation.

All MeV isolates from the MeV-HSV-1-coinfected cases were from the same genotype group (H1a). Some had identical 450-nt sequences. Due to incomplete epidemiological information for these cases, we were not able to determine whether the viruses with identical 450-nt sequences were in the same chain of transmission. Consistent with other reports^23–25^, all HSV-1 isolates from this study had 4–10 repeated GAG in the gG gene, and the number of tandem repeat sequences did not affect the topological structure of the phylogenetic tree^23^. Using the nucleic acid sequence of the whole gG gene, the phylogenetic analysis showed that most of the HSV-1 isolates identified in this study were in group A and that three isolates were in group B. This distribution is different from that found in European countries^26,27^. The nt 267–703 region in the HSV-1 gG gene has been identified as a recombination hotspot between groups A and B^23^. It has been noticed that the grouping of HSV-1 differs when different segments of the gG gene sequence are used for phylogenic analysis, suggesting that recombination has occurred within this region between the two groups^23,28^. Using sequence analysis and SimPlot, we did not find any evidence of recombination events in this region in the 37 HSV-1 isolates. In this study, we found 17 loci in the gG gene where the bases were completely different between group A and group B. More sequences are needed to determine whether these loci also serve as recombination hotspots.

Fatal cases of coinfection with MeV and other pathogens have been reported^12^. It is not clear whether MeV infection increases susceptibility to HSV-1 infection or triggers reactivation. Because of the high prevalence of HSV-1 in the population and the ability of HSV-1 to cause latent infections, the number of coinfection cases may be underestimated. Additional research is needed to investigate coinfections of MeV and other pathogens and to determine whether these co-infections affect the disease severity and/or require special treatment.

## Methods

### Ethics statement

The experiments and protocols in this study were screened and approved by the Ethical Review Committee of the Shanghai Municipal Centre for Disease Control and Prevention (Shanghai CDC). Clinical specimens, including throat swabs and sera were obtained from the routine measles virus surveillance programme of the Shanghai CDC. Sample collection was approved by either the patients or their parents with prior informed consent.

### Enrollment in patient and healthy groups and case classification

The demographic data of the patients were obtained from the China Information System for Disease Control and Prevention and the MSS. All met the definition of suspected measles cases based on the guidelines of the Shanghai Municipal Measles and Rubella Surveillance Program. The sera and throat swab samples used in this study were collected from suspected measles cases or healthy people by the Shanghai Municipal Measles and Rubella Surveillance Program and the Shanghai Municipal Surveillance Program for Population Immunization Rates and Vaccine Success Rate. Demographic data were obtained from the 2006–2015 Shanghai Statistical Yearbook.

In this study, the measles group consisted of cases confirmed by either MeV IgM testing or MeV RNA testing by real-time RT-PCR assay in 2015. The MeV and HSV-1 coinfection group included confirmed measles cases with HSV-1 infection that exhibited atypical CPE in infected cells after three passages. In addition, sera and throat swabs were collected from individuals who did not have clinical presentation or signs of measles at the time of sample collection to serve as controls (“healthy group”). The total numbers of sera samples in the measles, coinfection, and healthy groups were 741, 40 and 741, respectively, and the numbers of throat swab specimens collected for these three groups were 463, 40 and 192, respectively.

To obey ethical guidelines and ensure data protection, the serum and throat swab samples used in this study were collected according to the guidelines of the national and Shanghai municipal measles and rubella surveillance programs and did not involve human experimentation. This study strictly followed the relevant ethical requirements and strictly protected the data security and privacy of the participants.

### Detection of viral nucleic acids

Viral nucleic acid from throat swab samples or infected Vero/hSLAM cultures was extracted using the QIAamp Viral RNA/DNA Mini Kit (QIAGEN; Hilden, Germany) according to the manufacturer’s instructions. Viral nucleic acid was detected by real-time PCR/RT-PCR using a detection kit for MeV and rubella virus (RuV) (BioPerfectus Technologies, Taizhou, Jiangsu, China), human herpes virus type 6 (HHV6) and varicella-zoster virus (VZV) (Tianlong Biotechnology Co. Suzhou, Jiangsu, China) according to the manufacturer’s instructions. The detection of other viral febrile respiratory illnesses, such as human cytomegalovirus (CMV)^29^, Epstein-Barr virus (EB)^30^, human parvovirus B19 (B19)^31^, human adenovirus (Adv), human enterovirus (HEV)^32^ and herpes simplex virus type 1^33^, were carried out using the One Step PrimeScript™ RT-PCR Kit (Perfect Real Time) or Premix Ex Taq™ (Probe qPCR) (TaKaRa; Dalian, China) for RNA or DNA viruses as reported previously.

### Serological tests

Serological specimens were collected within 5 days after the onset of measles infection. Detection of MeV IgM was carried out using an IgM ELISA test kit (Haitai Biological Pharmaceutical Co.; Zhuhai, Guangdong, China). MeV IgG, HSV-1 IgM, and HSV-1 IgG were detected using reagent kits manufactured by Institute Virion/Serion GmbH (Würzburg, Germany). All procedures and the interpretation of the results were performed according to the manufacturer’s instructions. Specifically, if the results of two repeated experiments were consistent for the cases within the cut off value, it would be judged as negative.

### Virus isolation

The Vero/hSLAM cell line was used for MeV isolation from clinical specimens^11^. Vero/hSLAM cells were kindly shared by Dr. Yanagi at Kyushu University, Japan, through the WHO Global Measles and Rubella Laboratory Network (GMRLN). Procedures used for the maintenance of cells, sample handling and virus infection were performed as described in the Manual for the Laboratory-based Surveillance of Measles, Rubella, and Congenital Rubella Syndrome (WHO)^34^.

### Indirect immunofluorescence assay (IFA) and laser scanning confocal microscopy

A throat swab sample, SH13390, from a confirmed MeV and HSV-1 coinfected case was used to infect Vero/hSLAM cells, which was followed by ten cell passages. Infected cells from each passage were subjected to IFA. Briefly, the cells were washed with 0.01 M phosphate-buffered solution (PBS) and fixed with 4% polyoxymethylene solution (300 µL/well) at room temperature for 30 min, and they were then washed three times with PBS. The fixed cells were permeabilized with 0.5% Triton X-100 (Life Science, USA), washed with PBS, and blocked using 1% fetal bovine serum (FBS) in PBST (PBS plus 0.5% Tween 20) for 30 min at room temperature. To detect MeV and HSV-1 simultaneously, the cells were incubated with mixed diluted rabbit anti-MeV matrix (M) protein (Abcam, Shanghai, China) and mouse anti-HSV-1 ICP0 antibodies (Abcam, Shanghai, China) at 37 °C for 60 min. Subsequently, the cells were washed with PBST and incubated with mixed diluted fluorescently labeled antibodies at 37 °C for 60 min in the dark. The cell nuclei were stained with 1 μg/mL DAPI (Sigma, MO, USA). Images were obtained at 40× magnification with immersion oil by a Leica TCS SP8 laser scanning confocal microscope (Leica Microsystems; Wetzlar, Germany).

### Nucleotide sequencing

The genotyping of MeV was carried out as described previously^35^. In brief, a region of 676 nt near the C-terminus of the MeV N gene was amplified using the SuperScript™ III One-Step RT-PCR System with Platinum™ Taq High Fidelity DNA Polymerase (Invitrogen, Carlsbad, CA, USA). A 1075-bp region in the gG gene of HSV-1 was amplified with genotype-specific primers (HSV gG-F: 5′-GCTTTGTTTGCCGCTGTTTC-3′ and HSV gG-R: 5′-AAGTCGTGTGCTGTTTCTCC-3′) using the TaKaRa PCR Amplification Kit (TaKaRa) with the following conditions: one cycle of 94 °C for 5 min and 35 cycles of 94 °C for 45 s, 58 °C for 45 s and 72 °C for 70 s followed by one cycle of 72 °C for 7 min. The sequencing of the PCR products was performed at Sangon Biotech (Shanghai, China).

### Bioinformatics and statistics

The sequences were curated and assembled using Sequencher DNA Sequence Analysis Software (Gene Codes Corporation; Ann Arbor, MI). The sequence alignment and phylogenic analysis were performed using MEGA 6.0 and BioEdit software^36,37^. A chi-squared test and Wilcoxon nonparametric statistical test were performed using R^38^.

## Supplementary information

Dataset 1

## Data Availability

All data included in this study are available upon request by contact with the corresponding author or the first author.
