# Supplementary material for: The Detection and Characterization of Herpes Simplex Virus Type 1 in Confirmed Measles Cases
Source: Sci Rep. 2019 Sep 4;9:12785. doi: 10.1038/s41598-019-48994-5 (PMC6726758; doi:10.1038/s41598-019-48994-5)
Supplement: Supplementary file 1 — Dataset 1 [file 41598_2019_48994_MOESM1_ESM.pdf]

The Detection and Characterization of Herpes Simplex Virus Type 1 in Confirmed Measles Cases

Chongshan Li[1,2], Yunyi Li[2], Yuying Yang[2], Jing Wang[2], Caixia Zhu[3], Suwen Tang[2], Cong Pang[2], Wei Tang[2], Qiliang Cai[3], Zhi Li[2], Jiayu Hu[2], Xiaoxian Cui[2], Xi Zhang[2], Songtao Xu[4], Yan Zhang[4], Zhengan Yuan[5*], Yunwen Hu[1*], and Zhenghong Yuan[3*]

[1]Scientific Research Unit, Shanghai Public Health Clinical Center, Fudan University, Shanghai, 201508, People's Republic of China

[2]Division of Microbiology, Shanghai Municipal Center for Disease Control and Prevention, Shanghai, 200336, People's Republic of China

[3]MOE & MOH Key Laboratory of Medical Molecular Virology, School of Basic Medicine, Shanghai Medical College, Fudan University, Shanghai, 200032, People's Republic of China

[4]NHC Key Laboratory of Medical Virology and Viral Diseases (National Institute for Viral Disease Control and Prevention, Chinese Center for Disease Control and Prevention), Beijing, People's Republic of China.

[5]Shanghai Municipal Center for Disease Control and Prevention, Shanghai, 200336, People's Republic of China

***Corresponding authors**

Zhengan Yuan

1380, Zhongshan Road (W), Changning District, Shanghai, China

Tel: +8621 62758710

Fax: +8621 62756323

Email: yuanzhengan@scdc.sh.cn

Yunwen Hu

2901, Caolang Road, Jinshan District, Shanghai, China

Tel: +8621 37990333

Fax: +8621 57249023

Email: ywhu0117@126.com

Zhenghong Yuan

138, Shanghai Medical College Road, Xuhui District, Shanghai, China

Tel: +8621 64161928

Fax: +8621 64227201

Email: 13916306983@163.com

Supplementary Table S1. Value of IgG and IgM ELISA of individual cases coinfected with MeV and HSV-1 from 2006 to 2015 in Shanghai, China

Sample ID	IgM				IgG			
	MeV		HSV-1		MeV		HSV-1	
	OD Value	Judgement	OD Value	Judgement	OD Value	Judgement	OD Value	Judgement
SH08035	0.658	Pos	0.317	Neg	0.029	Neg	0.18	Neg
SH11300	0.114	Neg	0.083	Neg	0.009	Neg	0.149	Neg
SH12538	0.815	Pos	0.388	Neg	0.057	Neg	0.114	Neg
SH13774	0.178	Pos	0.418	Neg	0.729	Neg	1.55	Pos
SH06064	0.472	Pos	0.531	Neg	1.168	Pos	0.175	Neg
SH151187	1.029	Pos	0.579	Neg	0.347	Pos	0.127	Neg
SH12244	0.734	Pos	0.426	Neg	0.307	Neg	0.716	Pos
SH13017	0.87	Pos	0.108	Neg	0.115	Neg	0.328	Pos
SH13143	0.811	Pos	0.572	Neg	0.293	Neg	0.219	Pos
SH13176	0.349	Pos	0.13	Neg	0.021	Neg	0.821	Pos
SH13294	0.613	Pos	0.576	Neg	0.076	Neg	0.697	Pos
SH13770	0.767	Pos	0.145	Neg	0.057	Neg	0.17	Pos
SH15679	0.513	Pos	0.639	Neg	0.09	Neg	0.951	Pos
SH07040	0.892	Pos	0.51	Neg	0.909	Pos	0.767	Pos
SH11303	0.477	Pos	0.393	Neg	0.819	Pos	0.953	Pos
SH12164	0.613	Pos	0.378	Neg	1.119	Pos	0.811	Pos
SH12354	0.415	Pos	0.415	Neg	0.802	Pos	0.721	Pos
SH12525	0.862	Pos	0.228	Neg	0.965	Pos	0.484	Pos
SH12532	0.183	Pos	0.344	Neg	0.392	Pos	1.273	Pos
SH12649	0.134	Neg	0.603	Neg	0.619	Pos	0.266	Pos
SH13027	0.74	Pos	0.21	Neg	0.88	Pos	0.587	Pos
SH13073	0.799	Pos	0.374	Neg	0.922	Pos	1.714	Pos
SH13390	0.437	Pos	0.314	Neg	1.015	Pos	0.603	Pos
SH13401	0.4	Pos	0.274	Neg	1.031	Pos	0.752	Pos
SH13577	0.941	Pos	0.369	Neg	0.862	Pos	0.563	Pos
SH13659	0.194	Pos	0.882	Neg	1.344	Pos	0.885	Pos
SH14354	0.507	Pos	0.662	Neg	0.35	Pos	0.333	Pos
SH14622	0.563	Pos	0.404	Neg	0.707	Pos	0.575	Pos
SH151195	0.302	Pos	0.346	Neg	1.407	Pos	0.256	Pos
SH15180	0.467	Pos	0.399	Neg	0.581	Pos	0.246	Pos
SH15214	0.397	Pos	0.091	Neg	0.738	Pos	0.376	Pos
SH15334	1.11	Pos	0.542	Neg	0.549	Pos	0.578	Pos
SH15370	0.021	Neg	0.041	Neg	0.489	Pos	0.485	Pos
SH12441	0.86	Pos	1.067	Pos	0.081	Neg	0.297	Pos
SH15185	0.433	Pos	2	Pos	0.178	Neg	0.221	Pos
SH06036	0.804	Pos	3.226	Pos	0.882	Pos	0.649	Pos
SH06076	0.2	Pos	1.197	Pos	1.011	Pos	0.228	Pos

SH09046	0.808	Pos	0.988	Pos	0.394	Pos	0.224	Pos
SH14297	0.994	Pos	1.081	Pos	0.364	Pos	0.849	Pos
SH15226	0.487	Pos	1.102	Pos	1.064	Pos	0.8	Pos

Note:

1. cut off value for MeV IgM : 0.147
2. cut off value for HSV-1 IgM : 0.66-0.89
3. cut off value for MeV IgG : 0.26-0.32
4. cut off value for HSV-1 IgG : 0.14-0.21

Supplementary Table S2. Sequence Information of MeV Isolates from patients coinfected with MeV and HSV-1 from 2006 to 2015 in Shanghai, China

Sample ID	Sequence_ID	GenBank accession number
SH04005	MVi/Shanghai.CHN/17.04/02	MN166369
SH06064	MVi/Shanghai.CHN/26.06/01	MN166370
SH06076	MVi/Shanghai.CHN/46.06/01	MN166371
SH09046	MVi/Shanghai.CHN/48.09/01	MN166372
SH11303	MVi/Shanghai.CHN/28.11/01	MN166373
SH12525	MVi/Shanghai.CHN/20.12/11	MN166374
SH12532	MVi/Shanghai.CHN/21.12/03	MN166375
SH12538	MVi/Shanghai.CHN/20.12/12	MN166376
SH12649	MVi/Shanghai.CHN/24.12/04	MN166377
SH12936	MVi/Shanghai.CHN/40.12/01	MN166378
SH13017	MVi/Shanghai.CHN/05.13/03	MN166379
SH13401	MVi/Shanghai.CHN/18.13/33	MN166380
SH13659	MVi/Shanghai.CHN/26.13/01	MN166381
SH13782	MVi/Shanghai.CHN/34.13/01	MN166382
SH13791	MVi/Shanghai.CHN/34.13/03	MN166383
SH14001	MVi/Shanghai.CHN/01.14/01	MN166384
SH14004	MVi/Shanghai.CHN/01.14/02	MN166385
SH14013	MVi/Shanghai.CHN/04.14/01	MN166386
SH14019	MVi/Shanghai.CHN/06.14/01	MN166387
SH14044	MVi/Shanghai.CHN/09.14/02	MN166388
SH14144	MVi/Shanghai.CHN/13.14/14	MN166389
SH14354	MVi/Shanghai.CHN/17.14/10	MN166390
SH15214	MVi/Shanghai.CHN/08.15/06	MN166391
SH151187	MVi/Shanghai.CHN/20.15/20	MN166392
SH12164	MVi/Shanghai.CHN/13.12/06	MN226847
SH13027	MVi/Shanghai.CHN/07.13/01	MN226848
SH13073	MVi/Shanghai.CHN/10.13/05	MN226849
SH13143	MVi/Shanghai.CHN/11.13/05	MN226850
SH13390	MVi/Shanghai.CHN/17.13/30	MN226851
SH14622	MVi/Shanghai.CHN/30.14/03	MN226852
SH15679	MVi/Shanghai.CHN/15.15/19	MN226853
SH13294	MVi/Shanghai.CHN/15.13/27	MN226854
SH15180	MVi/Shanghai.CHN/06.15/09	MN226855
SH15185	MVi/Shanghai.CHN/07.15/09	MN226856
SH15370	MVi/Shanghai.CHN/12.15/26	MN226857

Supplementary Table S3. Sequence Information of HSV-1 Isolates from patients coinfected with MeV and HSV-1 from 2006 to 2015 in Shanghai, China

Sample ID	Sequence ID	GenBank accession number
SH06064	SH06064-gG	MN166405
SH06076	SH06076-gG	MN166406
SH07040	SH07040-gG	MN166407
SH09046	SH09046-gG	MN166408
SH11300	SH11300-gG	MN166409
SH11303	SH11303-gG	MN166410
SH12164	SH12164-gG	MN166411
SH12244	SH12244-gG	MN166412
SH12354	SH12354-gG	MN166413
SH12525	SH12525-gG	MN166414
SH12532	SH12532-gG	MN166415
SH12538	SH12538-gG	MN166416
SH12649	SH12649-gG	MN166417
SH13017	SH13017-gG	MN166418
SH13027	SH13027-gG	MN166419
SH13073	SH13073-gG	MN166420
SH13176	SH13176-gG	MN166422
SH13294	SH13294-gG	MN166423
SH13390	SH13390-gG	MN166424
SH13401	SH13401-gG	MN166425
SH13143	SH13143-gG	MN166421
SH13577	SH13577-gG	MN166426
SH13659	SH13659-gG	MN166427
SH13770	SH13770-gG	MN166428
SH13774	SH13774-gG	MN166429
SH14297	SH14297-gG	MN166430
SH14354	SH14354-gG	MN166431
SH14622	SH14622-gG	MN166432
SH15180	SH15180-gG	MN166433
SH15185	SH15185-gG	MN166434
SH15214	SH15214-gG	MN166435
SH15226	SH15226-gG	MN166436
SH15334	SH15334-gG	MN166437
SH15370	SH15370-gG	MN166438
SH15679	SH15679-gG	MN166439
SH151187	SH151187-gG	MN166440
SH151195	SH151195-gG	MN166441
